# A Large‐Scale Full *GBA1* Gene Screening in Parkinson's Disease in the Netherlands

**DOI:** 10.1002/mds.28112

**Published:** 2020-07-02

**Authors:** Jonas M. den Heijer, Valerie C. Cullen, Marialuisa Quadri, Arnoud Schmitz, Dana C. Hilt, Peter Lansbury, Henk W. Berendse, Wilma D.J. van de Berg, Rob M.A. de Bie, Jeffrey M. Boertien, Agnita J.W. Boon, M. Fiorella Contarino, Jacobus J. van Hilten, Jorrit I. Hoff, Tom van Mierlo, Alex G. Munts, Anne A. van der Plas, Mirthe M. Ponsen, Frank Baas, Danielle Majoor‐Krakauer, Vincenzo Bonifati, Teus van Laar, Geert J. Groeneveld

**Affiliations:** ^1^ Centre for Human Drug Research Leiden The Netherlands; ^2^ Leiden University Medical Center Leiden The Netherlands; ^3^ Lysosomal Therapeutics Inc Cambridge Massachusetts USA; ^4^ Erasmus Medical Center Rotterdam The Netherlands; ^5^ Janssen Vaccines and Prevention Leiden The Netherlands; ^6^ GenomeScan B.V. Leiden The Netherlands; ^7^ Amsterdam University Medical Centers Amsterdam The Netherlands; ^8^ University Medical Center Groningen Groningen The Netherlands; ^9^ Haga Teaching Hospital The Hague The Netherlands; ^10^ St. Antonius Ziekenhuis Nieuwegein The Netherlands; ^11^ Spaarne Gasthuis Haarlem The Netherlands; ^12^ Alrijne Ziekenhuis Leiden The Netherlands; ^13^ Meander Medical Center Amersfoort The Netherlands

**Keywords:** familial aggregation, GBA sequencing, genetic risk factor, glucocerebrosidase, heredity

## Abstract

**Background:**

The most common genetic risk factor for Parkinson's disease known is a damaging variant in the *GBA1* gene. The entire *GBA1* gene has rarely been studied in a large cohort from a single population. The objective of this study was to assess the entire *GBA1* gene in Parkinson's disease from a single large population.

**Methods:**

The *GBA1* gene was assessed in 3402 Dutch Parkinson's disease patients using next‐generation sequencing. Frequencies were compared with Dutch controls (n = 655). Family history of Parkinson's disease was compared in carriers and noncarriers.

**Results:**

Fifteen percent of patients had a *GBA1* nonsynonymous variant (including missense, frameshift, and recombinant alleles), compared with 6.4% of controls (OR, 2.6; *P* < 0.001). Eighteen novel variants were detected. Variants previously associated with Gaucher's disease were identified in 5.0% of patients compared with 1.5% of controls (OR, 3.4; *P* < 0.001). The rarely reported complex allele p.D140H + p.E326K appears to likely be a Dutch founder variant, found in 2.4% of patients and 0.9% of controls (OR, 2.7; *P* = 0.012). The number of first‐degree relatives (excluding children) with Parkinson's disease was higher in p.D140H + p.E326K carriers (5.6%, 21 of 376) compared with p.E326K carriers (2.9%, 29 of 1014); OR, 2.0; *P* = 0.022, suggestive of a dose effect for different *GBA1* variants.

**Conclusions:**

Dutch Parkinson's disease patients display one of the largest frequencies of *GBA1* variants reported so far, consisting in large part of the mild p.E326K variant and the more severe Dutch p.D140H + p.E326K founder allele. © 2020 The Authors. *Movement Disorders* published by Wiley Periodicals LLC. on behalf of International Parkinson and Movement Disorder Society.

The most common genetic risk factor known to date for Parkinson's disease (PD) is a damaging variant in the *GBA* gene (*GBA1*), encoding the lysosomal glucocerebrosidase enzyme.^1^ To avoid confusion with the nonlysosomal genes *GBA2* and *GBA3*, the *GBA* gene is also referred to as *GBA1*. In most populations, 4%‐12% of PD patients carry a heterozygous *GBA1* variant and in Ashkenazi Jewish PD patients this is approximately 20%.^2^, ^3^ The risk of PD in *GBA1* variant carriers is increased by an estimated overall 2‐ to 7‐fold (odds ratios [ORs]).^2^, ^3^, ^4^, ^5^ Rare homozygous or compound heterozygous *GBA1* variants can cause the autosomal‐recessive lysosomal storage disorder Gaucher's disease (GD). More than 400 variants have been reported to be associated with GD,^6^, ^7^ and all these alleles are potential risk factors for developing PD.

Full *GBA1* gene sequencing is essential to unambiguously identify gene variants, considering a long tail of rare variants or even population‐specific variants.^3^, ^4^, ^8^ Nevertheless, rarely the entire *GBA1* gene has been sequenced in a large cohort from a single population. Here, we report such a large‐scale *GBA1* screening performed in the Netherlands in the framework of a large program aimed at identifying patients with *GBA1* variants for a clinical trial targeting the *GBA1* mechanism. We sequenced the *GBA1* entire open‐reading frame (ORF) in 3402 people with PD living in the Netherlands. Variant frequency was compared with an existing Dutch control cohort (n = 655). Family history of PD was assessed in a subset of patients with the most common variants to compare familial aggregation.

## Materials and Methods

### Participants

PD patients were included in the Netherlands between April 2017 and March 2018 (see supplementary data for details). Age at diagnosis of ≤50 years was considered early onset, and > 50 years was considered late‐onset PD.

This study was approved by an independent ethics committee. Written informed consent was obtained from all participants according to the Declaration of Helsinki.

An independent Dutch study of 655 patients with abdominal aortic aneurysms was used for comparison (see supplementary data), using whole‐exome sequencing (WES) data (average *GBA1* coverage was 101 times). Data regarding the presence of neurological disease were unavailable.

### Genotyping

Saliva was obtained from patients using Oragene DNA OG‐500 tubes (DNA Genotek). DNA isolation, next‐generation sequencing (NGS), and data analysis was performed by GenomeScan B.V., Leiden, the Netherlands. Primers were selected to unambiguously sequence the functional *GBA1* gene and not the pseudogene, using long‐range polymerase chain reaction (PCR). In a post hoc experimental setup using long‐read sequencing with the PacBio Sequel system, phasing was assessed in 3 samples. See supplementary material for methodological details, including validation of a subset using Sanger sequencing.

Historically, *GBA1* variants have been described based on the amino acid position excluding the 39‐residue signal sequence at the start (also known as “allelic nomenclature”). Both the Human Genome Variation Society recommended nomenclature, and the allelic nomenclature is given (NCBI Reference Sequence: NM_000157.3). If an allele contained more than 1 exonic variant, this is referred to as a complex allele.

Genotypes were classified into 4 categories based on clinical associations using the Human Gene Mutation Database^7^: (1) Gaucher's disease associated (GD), (2) Parkinson's disease associated (PD), (3) synonymous, or (4) novel. If a subject had both a known and a novel variant, the genotype was considered novel. See supplementary data for details.

All variants that were 6 nucleotides or closer to a splice site were assessed with 4 in silico splicing programs implemented in Alamut (Alamut Visual version 2.13; see supplementary data).

A 2‐step cross‐validation was performed to assess risk of both false‐positive and false‐negative results when using WES (see supplementary data).

### Family History

All patients with the *GBA1* p.D140H + p.E326K, p.E326K, p.N370S, or p.L444P variants and a random subset of patients who did not carry *GBA1* variants as per our methods and variant selection criteria (henceforth referred to as *GBA1* wild type) were given a questionnaire to assess familial aggregation of PD and to assess a possible founder location of the p.D140H + p.E326K complex allele. See supplementary material for details.

### Statistical Analysis

Fisher's exact test was used for categorical variables and the Mann‐Whitney *U* test for continuous variables. Significance was flagged at *P* < 0.05. ORs were calculated with a 95% CI. IBM SPSS Statistics 25 software was used.

## Results

In total, 3638 PD patient samples were included, of which 3402 could be genotyped. Of the remaining 236 samples, no DNA could be extracted or PCR failed. Demographics can be found in Supplementary Table 1. Eighty‐one percent of patients were recruited through referral by a neurologist.

### Sequencing

Average coverage was 2703 times (Supplementary Fig. 1). The subset of samples used in the Sanger sequencing validation were all confirmed (see supplementary data).

### *GBA1* Variants

All *GBA1* exonic and splice‐site variants are listed in Table 1, including frequency comparison between PD patients and controls. In short, the total PD cohort had 15.0% nonsynonymous variants (including missense, frameshift, and recombinant alleles) versus 6.4% in controls (OR, 2.6; 95% CI, 1.9–3.6; *P* < 0.001). For GD variants observed in patients (5.0%) versus controls (1.5%), the OR was 3.4 (95% CI, 1.8–6.5; *P* < 0.001) and for the PD variants observed in patients (9.3%) versus controls (4.4%), the OR was 2.2 (95% CI, 1.5–3.3; *P* < 0.001).

**TABLE 1:** Listing of all found exonic and splice‐site variants, including specifications [Color table can be viewed at wileyonlinelibrary.com]

Genotype information							Cohorts			
Position Chr 1	cDNA	rsID	Exon	Protein	Allelic name	Clinical	PD patients	Control	OR	*P*
(GRCh37/hg19)	NM_000157.3			NP_000148.2		association	% (n)	% (n)	(95% CI)	
							(n = 3402)	(n = 655)		
Heterozygous (simple and complex)										
155210876:C	c.26_27del	‐	1	p.(Glu9GlyfsTer8)	E‐30Gfs*8	Novel	0.0 (1)	0 (0)	NA	NA
155210492:G	c.44T > C	‐	2	p.(Leu15Ser)	L‐24S	Novel	0.0 (1)	0 (0)	NA	NA
155210492:G	c.44T > C	‐	2	p.[(Leu15Ser;Ser16Gly)]	L‐24S + S‐23G	Novel	0.0 (1)	0 (0)	NA	NA
155210490:C	c.46A > G		2			Novel				
155210441:C	c.95A > G	‐	2	p.(Gln32Arg)	Q‐7R	Novel	0.0 (1)	0 (0)	NA	NA
155209813:T	c.171C > A	‐	3	p.(Cys57Ter)	C18*	Novel	0.0 (1)	0 (0)	NA	NA
155209752:A	c.232C > T	rs146774384	3	p.(Arg78Cys)	R39C	Novel	0.0 (1)	0 (0)	NA	NA
155209732:AC	c.251_252insC	‐	3	p.(Ser84ArgfsTer15)	S45Rfs*15	Novel	0.0 (1)	0 (0)	NA	NA
155208421:A	c.475C > T	rs397515515	5	p.(Arg159Trp)	R120W	GD	0.1 (5)	0 (0)	NA	NA
155208361:G	c.535G > C	rs147138516	5	p.[(Asp179His;Glu365Lys)]	D140H + E326K	GD	2.4 (82)	0.9 (6)	2.7	**0.012**
155206167:T	c.1093G > A	rs2230288	8						(1.2‐6.1)	
155208060:T	c.626G > A	‐	6	p.(Arg209His)	R170H	Novel	0.0 (1)	0 (0)	NA	NA
155208001:T	c.685G > A	‐	6	p.(Ala229Thr)	A190T	GD	0.0 (1)	0 (0)	NA	NA
155207965:T	c.721G > A	rs398123534	6	p.(Gly241Arg)	G202R	GD	0.0 (1)	0 (0)	NA	NA
155207367:T	c.764T > A	rs74500255	7	p.(Phe255Tyr)	F216Y	GD	0.0 (1)	0 (0)	NA	NA
155207266:T	c.865G > A	‐	7	p.(Gly289Ser)	G250S	Novel	0.0 (1)	0 (0)	NA	NA
155207249:C	c.882T > G	rs367968666	7	p.(His294Gln)	H255Q	GD	0.1 (2)	0 (0)	NA	NA
155207235:G	c.896T > C	‐	7	p.(Ile299Thr)	I260T	GD	0.1 (2)	0 (0)	NA	NA
155206172:G	c.1088T > C	‐	8	p.(Leu363Pro)	L324P	GD	0.0 (1)	0.2 (1)	0.2	0.297
									(0.0–3.1)	
155206170:T	c.1090G > A	rs121908305	8	p.(Gly364Arg)	G325R	GD	0.0 (1)	0 (0)	NA	NA
155206167:T	c.1093G > A	rs2230288	8	p.(Glu365Lys)	E326K	PD	6.3 (213)	2.6 (17)	2.5	**<.001**
									(1.5–4.1)	
155206158:A	c.1102C > T	rs374306700	8	p.(Arg368Cys)	R329C	GD	0.1 (2)	0 (0)	NA	NA
155206101:C	c.1159T > G	‐	8	p.(Trp387Gly)	W348G	GD	0.0 (1)	0 (0)	NA	NA
155206093:G	c.1167G > C	‐	8	p.(Gln389His)	Q350H	Novel	0.0 (1)	0.2 (1)	0.2	0.297
									(0.0–3.1)	
155206037:A	c.1223C > T	rs386626586	8	p.(Thr408Met)	T369M	PD	2.5 (86)	1.8 (12)	1.4	0.332
									(0.8–2.6)	
155205634:C	c.1226A > G	rs76763715	9	p.(Asn409Ser)	N370S	GD	0.9 (30)	0.3 (2)	2.9	0.151
									(0.7–12.2)	
155205619:C	c.1241T > G	‐	9	p.(Val414Gly)	V375G	Novel	0.0 (1)	0 (0)	NA	NA
155205605:A	c.1255G > T	‐	9	p.(Asp419Tyr)	D380Y	GD	0.0 (1)	0 (0)	NA	NA
155205581:T	c.1279G > A	rs149171124	9	p.(Glu427Lys)	E388K	PD	0.1 (3)	0 (0)	NA	NA
155205568:C	c.1292A > G	‐	9	p.(Asn431Ser)	N392S	PD	0.0 (1)	0 (0)	NA	NA
155205518:G	c.1342G > C	rs1064651	9	p.(Asp448His)	D409H	GD	0.0 (1)	0 (0)	NA	NA
155205043:G	c.1448T > C	rs421016	10	p.(Leu483Pro)	L444P	GD	0.6 (21)	0 (0)	NA	**0.037**
155205016:A	c.[1475A > T; 1474G > C]	‐	10	p.(Asp492Leu)	D453L	Novel	0.1 (4)	0 (0)	NA	NA
155205017:G			10		(D453V + D453H)					
155204996:T	c.1495G > A	‐	10	p.(Val499Met)	V460M	GD	0.0 (1)	0 (0)	NA	NA
155204986:G	c.1505G > C	‐	10	p.(Arg502Pro)	R463P	GD	0.1 (2)	0.2 (1)	0.4	0.410
									(0.0–4.2)	
155204829:A	c.1568C > T	‐	11	p.(Ser523Leu)	S484L	Novel	0.0 (1)	0 (0)	NA	NA
155204818:T	c.1579T > A	‐	11	p.(Ser527Thr)	S488T	PD	0.0 (1)	0 (0)	NA	NA
155204811:C	c.1586A > G	‐	11	p.(His529Arg)	H490R	Novel	0.0 (1)	0 (0)	NA	NA
Likely recombinant alleles										
155207210:A,	c.924C > T,	—	7	p.(Leu307=),	L268=, S271G, D409H	Novel	0.0 (1)	0 (0)	NA	NA
155207203:C,	c.931A > G,	—	7	p.(Ser310Gly),						
			9							
			9		D409H, L444P, A456P, V460=(a.k.a. Rec*TL*)	GD	0.0 (1)	0 (0)	NA	NA
			10							
155205008:G,	c.1483G > C,	—	10	p.(Ala495Pro),						
			10							
			10		L444P, A456P, V460=(a.k.a. Rec*Ncil*)	GD	0.1 (4)	0 (0)	NA	NA
			10							
			10							
Homozygous or compound heterozygous (variant details in listing above)										
				p.[(Leu363Pro)];[(Thr408Met)]	L324P / T369M	GD / PD	0.0 (1)	0 (0)	NA	NA
				p.[(Asp179His;Glu365Lys)]; [(Thr408Met)]	D140H + E326K / T369M	GD / PD	0.0 (1)	0 (0)	NA	NA
				p.[(Asp179His;Glu365Lys)]; [(Glu365Lys)]	D140H + E326K / E326K	GD / PD	0.0 (1)	0 (0)	NA	NA
				p.[(Glu365Lys)];[(Thr408Met)]	E326K / T369M	PD / PD	0.1 (4)	0 (0)	NA	NA
				p.[(Glu365Lys)];[(Glu365Lys)]	E326K / E326K	PD / PD	0.2 (6)	0 (0)	NA	NA
				p.[(Thr408Met)];[(Thr408Met)]	T369M / T369M	PD / PD	0.0 (1)	0 (0)	NA	NA
Uncertain phasing (variant details in listing above)										
155210424:T, …	c.112T > A, …	—, …	2, …	p.(Ser38Thr)(;)(Thr408Met)	S‐1T, T369M	Novel, PD	0.0 (1)	0 (0)	NA	NA
				p.(Gln32Arg)(;)(Asn409Ser)	Q‐7R, N370S	Novel, GD	0.0 (1)	0 (0)	NA	NA
				p.[(Asp179His;Glu365Lys)](;)(Val498=)	D140H + E326K, V459=	GD, Syn	0.0 (1)	0 (0)	NA	NA
…, 155204793:T	…, c.1604G > A	…, rs80356773	…, 11	p.[(Asp179His;Glu365Lys)](;)Arg535His)	D140H + E326K, R496H	GD, GD	0.0 (1)	0 (0)	NA	NA
				p.(Arg209His)(;)(Glu365Lys)	R170H, E326K	Novel, PD	0.0 (1)	0 (0)	NA	NA
				p.[(Glu365Lys)];[(Thr408Met)](;)(Leu483Pro)	E326K / T369M, L444P	PD / PD, GD	0.0 (1)	0 (0)	NA	NA
…, 155205574:T	…, c.1286G > A	…, ‐	…, 9	p.(Glu365Lys)(;)(Gly429Glu)	E326K, G390E	PD, Novel	0.0 (1)	0.2 (1)	0.2	0.297
									(0.0–3.1)	
				p.(Glu365Lys)(;)(Val498=)	E326K, V459=	PD, Syn	0.0 (1)	0 (0)	NA	NA
				p.(Glu365Lys)(;)(Val499=)	E326K, V460=	PD, Syn	0.0 (1)	0 (0)	NA	NA
				p.(Thr408Met)(;)(Asp492Leu)	T369M, D453L	PD, Novel	0.0 (1)	0 (0)	NA	NA
				p.(Thr408Met)(;)(Leu483Pro)	T369M, L444P	PD, GD	0.1 (3)	0 (0)	NA	NA
				p.(Asn409Ser)(;)(Leu483Pro)	N370S, L444P	GD, GD	0.0 (1)	0 (0)	NA	NA
Synonymous										
155209816:A	c.168C > T	rs145773486	3	p.(Val56=)	V17=	Syn	0 (0)	0.2 (1)	NA	0.161
155209684:T	c.300G > A	—	3	p.(Thr100=)	T61=	Syn	0.0 (1)	0 (0)	NA	NA
155208422:A	c.474C > T	rs147411159	5	p.(Ile158=)	I119=	Syn	0.1 (5)	0 (0)	NA	NA
155208389:T	c.507C > A	—	5	p.(Ile169=)	I130=	Syn	0.0 (1)	0 (0)	NA	NA
155208350:T	c.546G > A	—	5	p.(Gln182=)	Q143=	Syn	0.0 (1)	0 (0)	NA	NA
155207990:T	c.696G > A	rs375731497	6	p.(Gly232=)	G193=	Syn	0.0 (1)	0.2 (1)	0.2	0.297
									(0.0‐3.1)	
155207984:A	c.702G > T	—	6	p.(Gly234=)	G195=	Syn	0.0 (1)	0 (0)	NA	NA
155206111:A	c.1149C > T	—	8	p.(Gly383=)	G344=	Syn	0.0 (1)	0 (0)	NA	NA
155206036:T	c.1224G > A	rs138498426	8	p.(Thr408=)	T369=	Syn	0.1 (2)	0 (0)	NA	NA
155205018:A	c.1473C > T	rs149257166	10	p.(Pro491=)	P452=	Syn	0.0 (1)	0 (0)	NA	NA
155204997:A	c.1494C > T	rs371779859	10	p.(Val498=)	V459=	Syn	0.1 (3)	0 (0)	NA	NA
155204994:G	c.1497G > C	rs1135675	10	p.(Val499=)	V460=	Syn	0.0 (1)	0 (0)	NA	NA
										
Splice site (distance of 6 nucleotides or less)										
155207374:T	c.762‐5G > A	—	*Intr*.	—	—	Novel	0.0 (1)	0 (0)	NA	NA
155206264:A	c.1000‐4G > T	—	*Intr*.	—	—	Novel	0 (0)	0.2 (1)	NA	0.161
Exonic variants (details above) fulfilling splice‐site criteria (variant [distance]) — see Supplementary Table 4 for splicing prediction: p.E‐30Gfs*8 (1), p.S‐1T (4), p.F216Y (3), p.T369= (1), p.T369M (2), p.N370S (2), p.R463P (1)										
Grouped comparisons										
All Novel genotypes							0.7 (23)	0.3 (2)	1.5	0.788
									(0.4–4.9)	
All PD genotypes (p.E326K, p.T369M, p.E388K, p.S488T, p.N392S)							9.3 (317)	4.4 (29)	2.2	**<0.001**
									(1.5–3.3)	
All GD genotypes							5.0 (170)	1.5 (10)	3.4	**<0.001**
									(1.8‐–6.5)	
Total non‐synonymous							15.0 (510)	6.4 (42)	2.6	**<0.001**
									(1.9–3.6)	

GD, Gaucher's disease; PD, Parkinson's disease; syn, synonymous; NA, not applicable; Intr., intronic.

The sixth column “allelic name” contains the annotation historically used in Gaucher's disease literature, excluding the 39–amino acid signaling peptide. All genotype frequencies are compared with the abdominal aortic aneurysm control cohort, ORs are given with the 95% CIs and a *P* value. A *P* < 0.05 is given in **boldface**, and the rows of these genotypes are filled gray. OR could not be calculated if frequency was 0 in either group. If 6 cases or less were affected in patients and zero in controls, *P* value is set to NA. The coding (or sense) strand for *GBA1* is the reverse strand of the DNA (as opposed to the forward strand). The chromosome position and nucleotide reflect the forward strand, whereas the cDNA annotation indicates the variant on the coding strand, which is in this case the reverse strand, and therefore these are complementary. Both intronic splice‐site variants were predicted not to affect splicing (see supplementary material) and were therefore not included in the overall analysis.

In total, 19 GD variants, 5 PD variants, 12 synonymous variants, and 18 novel variants were identified. In 1 sample with p.D140H + p.E326K, phasing was confirmed using PacBio sequencing. See supplementary data for a further description of variants found. Supplementary Table 3 contains a variant frequency comparison with data from GoNL^9^ and GnomAD^10^, ^11^ for reference; however, methodology in these cohorts was not dedicated to *GBA1* sequencing.

No intronic variants were assessed to have a possible effect on splicing (Supplementary Table 4).

### Control Cohorts Cross‐Validation

In the control cohort, 42 samples had a nonsynonymous *GBA1* variant detected using WES that could be tested with our NGS protocol. Using NGS, 4 control samples were detected to be false‐positive, and 3 samples were partially false‐negative (for p.D140H in a p.D140H + E326K complex allele). Conversely, after rerunning 48 GBA‐PD samples with WES, 1 false‐negative was detected. See supplementary data for details.

### Demographics Based on *GBA1* Status

Demographics are given in Supplementary Table 1, divided over whether subjects carried a nonsynonymous variant. A larger portion of carriers had early‐onset PD (27.2%) compared with noncarriers (18.2%), *P* < 0.001. Conversely, of all subjects with early onset, 20.1% had a *GBA1* variant, compared with 13.1% in those with late onset (*P* < 0.001).

### GBA Variants and Familial Aggregation of PD

A questionnaire was completed by 180 carriers of p.E326K, 24 carriers of p.N370S, 28 carriers of p.L444P (including 4 complex and 3 recombinant alleles), 73 carriers of p.D140H + p.E326K, and 135 *GBA1* wild types. Combining all carriers, 3.6% of all siblings and parents combined had PD compared with 2.0% in siblings and parents of noncarriers (OR, 1.8; 95% CI, 1.0–3.2; *P* = 0.043). None of the children developed PD, probably because of the present younger age, so these were excluded from analysis of first‐degree relatives (Supplementary Table 2). Supplementary Figure 2 depicts the total number of first‐degree relatives (excluding children) per variant type and the percentage of these relatives with PD. A variant dose effect was seen (see supplementary data for details).

### Founder Location p.D140H + p.E326K

Supplementary data and Supplementary Figure 3 show a heat map of descent of grandparents of p.D140H + p.E326K carriers, visually suggesting (no formal statistical testing) the northern Netherlands as a possible founder location for this complex allele.

## Discussion

To our knowledge, this study is the largest cohort known to date from a single country that has had full gene *GBA1* sequencing in PD patients. A total of 15.0% of all patients had nonsynonymous *GBA1* variants, which is the highest prevalence reported to date in a non‐Ashkenazi Jewish population. The relatively high prevalence of the population‐specific p.D140H + p.E326K complex allele and the long tail of rare variants, including 18 novel variants, highlight the importance of sequencing the full *GBA1* ORF. Identifying all these variants will strengthen our understanding of the effect of *GBA1* variants, and it facilitates recruitment for the upcoming *GBA1*‐targeted trials, hopefully resulting in a first disease‐modifying drug for PD.^12^

Comparing different countries,^3^, ^4^, ^8^, ^13^, ^14^, ^15^, ^16^, ^17^, ^18^, ^19^, ^20^, ^21^, ^22^, ^23^, ^24^, ^25^, ^26^ the p.E326K variant is reported most frequently in the Netherlands (present study) and Scandinavian countries.^20^, ^24^ Table 2 compares the most common *GBA1* variants and the p.D140H + p.E326K complex allele in large PD cohorts from single countries that performed full *GBA1* ORF sequencing. Swedish^24^ and Russian^15^ cohorts were included despite selective sequencing because of their size to compare the p.E326K variant. This overview shows the near‐exclusive appearance of p.D140H + p.E326K in the Netherlands. The p.D140H + p.E326K complex allele has only sporadically been reported, once in GD,^27^, ^28^ sporadically in PD^4^, ^29^ and once in Lewy body dementia.^30^

**TABLE 2:** International comparison of Parkinson's disease cohorts that performed full *GBA1* gene sequencing, sorted based on total percent of *GBA1* variant carriers [Color table can be viewed at wileyonlinelibrary.com]

International comparison of total and common GBA1 variants in Parkinson's disease cohorts								
	PD (n)	*GBA1* (%)	E326K	T369M	N370S	L444P	D140H + E326K	Other
Ashkenazi Jewish	735	18.0	1.6	0	11.8	0.3	0	4.2
**This cohort (NL)**	**3402**	**15.0**	**6.7**	**2.5**	**0.9**	**0.6**	**2.5**	**1.8**
France	1130	12.5	4.2	1.5	2.9	1	0.1	2.7
Colombia	131	12.2	1.5	0	2.3	2.3	0	6.1
Norway	442	12.0	6.6	3.6	0.2	1.4	0	0.5
Spain	532	11.7	3	0.9	0.9	2.4	0	4.3
United States	1369	11.6	5	2.2	1.3	1.2	0.1	1.9
United Kingdom	1893	11.1	4.5	1.8	0.6	1.6	0.1	2.4
Eastern Canada	225	11.1	1.8	4.9	0.9	1.8	0	1.8
Belgium	266	9.8	4.1	1.1	1.1	1.5	0.4	1.5
Japan	534	9.4	0	0	0	4.1	0	5.2
New Zealand	229	9.2	4.8	3.1	0.4	0	0.4	0.9
Sweden	1625	8.3	5.8	N/A	0.4	2.2	N/A	N/A
Peru	471	7.2	1.1	0.6	0.2	2.8	0	1.8
Russia	762	6.6	2.4	2.5	0.5	1.1	N/A	N/A
Greece	172	6.4	0.6	0	0	1.2	0	4.7
Portugal	230	6.1	0.9	0.9	2.2	1.3	0	0.9
Korea	277	6.1	0	0	0	0.7	0	5.4
North Africa	194	4.6	0.5	1.0	1.0	1.5	0	0.5

PD, Parkinson's disease; NL, the Netherlands; N/A, not applicable.

All variant frequencies are given in percentages. Sweden and Russia performed selective sequencing. France is a European study, with 89% of subjects from France. North Africa is primarily Algeria, but also Morocco, Tunisia, and Libya. References: Ashkenazi Jewish (1), Netherlands (current study), France (2), Colombia (3), Norway (4), Spain (5), United States (6), United Kingdom (7), eastern Canada (8), Belgium (9), Japan (10), New Zealand (11), Sweden (12), Peru (3), Russia (13), Greece (14), Portugal (15), Korea (16), and north Africa (17).

Intronic splice‐site variants have rarely been systematically assessed previously,^17^, ^23^; however, these do not seem to play a role in GBA‐PD pathology in our Dutch cohort.

The importance of adequate genotyping methodology when sequencing *GBA1* was once more confirmed. In the control cohort, the *GBA1* variants were reassessed with NGS, which identified 4 false‐positive p.L444P variants in WES. Also, 3 p.D140H variants were falsely not identified in 3 samples that also carried the p.E326K variant. The performance of the hybridization capture panel was lower over the p.D140H region, reflected in local lower coverage. Combined with a possible allelic imbalance for this specific variant, in which the amplification prefers the wild‐type allele over the p.D140H allele, this could explain the false‐negative output. Therefore, caution is advised when using *GBA1* data generated using a methodology not specifically designed for *GBA1* sequencing (including databases like ExAC or gnomAD).

Because the p.E326K and p.T369M variants do not cause Gaucher's disease, these have long been termed polymorphisms. However, it has been shown in meta‐analyses that these variants do confer an increased risk of developing PD (OR, 1.99 for p.E326K and 1.74 for p.T369M)^31^, ^32^, ^33^ and therefore, despite not causing GD, should not be considered neutral polymorphisms.

Of all participants diagnosed with PD at 50 years of age or younger, 20.1% had a *GBA1* variant. In clinical practice, when genetic testing is performed in early‐onset PD, *GBA1* is not always included. Because of the high prevalence of *GBA1* variants in early‐onset PD, it deserves consideration to include this in the screening, although the predictive value of a *GBA1* variant for offspring is still limited.

*GBA1* variant carriers have a larger frequency of a positive family history for Parkinson's disease^4^, ^5^, ^34^ compared with noncarriers. In the current study, carriers of p.D140H + p.E326K had significantly more first‐degree relatives with PD compared with p.E326K carriers. This implies a dose effect of variant severity in familial aggregation. However, it did not reach statistical significance for other variant types, likely because of the rarity of these variants.

The current study has some limitations. Because our NGS method used short‐read sequencing, phasing of multiple variants could not be determined, unless these were within approximately 500 base pairs of each other. However, for a single p.D140H + p.E326K sample phasing was confirmed using PacBio, and p.D140H was never seen without p.E326K. A recombinant gene could be identified if the long‐range PCR resulted in 2 distinct peaks on the Fragment Analyzer. See supplementary data for a further discussion of possible limitations.

In conclusion, this study is a successful example of how to ascertain and genotype a large cohort of patients with PD within a short time frame, which is relevant for progressing clinical trials aimed at developing personalized treatments.

The Dutch PD population appears to have a relatively large number of *GBA1* variant carriers, consisting mostly of the mild p.E326K variant and the likely more severe Dutch p.D140H + p.E326K complex allele, with a possible founder effect in the northern part of the Netherlands. In total, 18 novel *GBA1* variants were detected. *GBA1* variant carriers had a younger age at onset and a higher chance of a positive family history for PD, with a trend toward a dose effect based on clinical association of the variant.

## Authors’ Roles

1) Research project: A. Conception, B. Organization, C. Execution;

2) Statistical Analysis: A. Design, B. Execution, C. Review and Critique;

3) Manuscript: A. Writing of the first draft, B. Review and Critique.

Jonas M. den Heijer: 1A, 1B, 1C, 2A, 2B, 2C, 3A, 3B; Valerie C. Cullen: 1A,1B, 1C, 2C, 3B; Marialuisa Quadri: 1C, 2A, 2C, 3A, 3B; Arnoud Schmitz: 1A, 1B, 1C, 2C, 3B; Dana C. Hilt: 1A, 1B, 1C, 2C, 3B; Peter Lansbury: 1A, 1B, 1C, 2C, 3B; Henk W. Berendse: 1B, 1C, 2C, 3B; Wilma D.J. van de Berg: 1B, 1C, 2C, 3B; Rob M.A. de Bie: 1B, 1C, 2C, 3B; Jeffrey M. Boertien: 1C, 3B; Agnita J.W. Boon: 1B, 1C, 2C, 3B; M. Fiorella Contarino: 1B, 1C, 2C, 3B; Jacobus J. van Hilten: 1B, 1C, 2C, 3B; Jorrit I. Hoff: 1B, 1C, 2C, 3B; Tom van Mierlo: 1B, 1C, 2C, 3B; Alex G. Munts: 1B, 1C, 2C, 3B; Anne A. van der Plas: 1B, 1C, 2C, 3B; Mirthe M. Ponsen: 1B, 1C, 2C, 3B; Frank Baas: 1B, 1C, 2C, 3B; Danielle Majoor‐Krakauer: 1B, 1C, 2C, 3B; Vincenzo Bonifati: 1A, 1C, 2A, 2C, 3A, 3B; Teus van Laar: 1B, 1C, 2C, 3B; Geert J. Groeneveld: 1A, 1B, 1C, 2A, 2C, 3A, 3B.

## Financial Disclosures of all authors (for the preceding 12 months)

J.M. den Heijer: none.

Dr. V.C. Cullen was an employee and consultant of Lysosomal Therapeutics Inc. and owns stock options in the company.

Dr. M. Quadri: none.

A. Schmitz: none.

Dr. D.C. Hilt was an employee and consultant of Lysosomal Therapeutics Inc.

Dr. P. Lansbury was an employee and consultant of Lysosomal Therapeutics Inc.

Dr. H W. Berendse received research grants from the Michael J. Fox Foundation, the Netherlands Organisation for Health Research and Development (ZonMW), and the Netherlands Brain Foundation.

Dr. W.D.J. van de Berg: none.

Dr. De Bie reports grants from ZonMw (Dutch governmental fund for health research), grants from Parkinson Vereniging (Netherlands patient organization), grants from Stichting Parkinson Nederland (charitable foundation), and unrestricted research grants from GE Health, Medtronic, and Lysosomal Therapeutics (all paid to the institution).

J.M. Boertien: none.

Dr. A.J.W. Boon: none.

Dr. M.F. Contarino received support for advisory board from Medtronic (fees to institution). Consultancies: Medtronic (fees to institution), CHDR (fees to institution). Research support: Medtronic (to institution), AbbVie (to institution). Research support in kind from Global Kinetics Corporation. Travel support: Boston Scientific.

Dr. J.J. van Hilten reports grants from The Netherlands Organisation for Health Research and Development, The Netherlands Organisation for Scientific Research, Hoffmann‐La Roche, AbbVie, Lundbeck, Hersenstichting, Stichting Parkinson Fonds, Alkemade‐Keuls Foundation, and Centre of Human Drug Research.

Dr. J.I. Hoff: none.

Dr. T. van Mierlo: none.

Dr. A.G. Munts: none.

Dr. A.A. van der Plas: none.

Dr. M.M. Ponsen: none.

Dr. F. Baas is founder and shareholder of ComplementPharma, a company directed to the development of therapy based on complement therapeutics, and received funding from WAVE technologies, project related to testing CMT1A treatment in mice.

Dr. D. Majoor–Krakauer received funding from Lijf en Leven Foundation and the Jaap Schouten Foundation.

Dr. V. Bonifati discloses intellectual property rights: coinventor of a patent titled “Role for low density lipoprotein receptor‐related protein in progressive brain diseases.” He received honoraria from the International Parkinson and Movement Disorder Society, Springer, as section editor of *Current Neurology* and *Neuroscience Reports*, Elsevier as coeditor‐in‐chief of *Parkinsonism & Related Disorders*; grants from Stichting Parkinson Fonds (The Netherlands), Alzheimer Nederland, ZonMw (The Netherlands), under the aegis of the EU Joint Programme Neurodegenerative Disease Research (JPND), and Erasmus MC, Rotterdam.

Dr. T. van Laar received support for the advisory board from Britannia Pharm., Neuroderm, AbbVie; for speakers fees from Britannia Pharm. and AbbVie; grants from Weston Brain Institute.

Dr. G.J. Groeneveld: none.

## Supporting information


**Appendix** **S1:** Supplementary data
