# Supplementary material for: A Large‐Scale Full GBA1 Gene Screening in Parkinson's Disease in the Netherlands
Source: Mov Disord. 2020 Jul 2;35(9):1667–74. doi: 10.1002/mds.28112 (PMC7540512; doi:10.1002/mds.28112)
Supplement: Supplementary file 1 — Appendix S1: Supplementary data [file MDS-35-1667-s001.zip › MDS_28112_Den Heijer et al_GBA1 Genotyping_Brief Report_Supplementary material_14Mar20.docx]

# Supplementary data

## Supplementary Material and methods

**Participants**

PD patients were included using two recruiting methods: (1) Neurologists specialized in movements disorders from ten hospitals in the Netherlands informed all their patients about the study; and (2) patients were informed through other media (e.g. patient associations). Patients were asked to confirm that the diagnosis of PD was made by a neurologist and at what age the patient had been diagnosed.

The independent Dutch study of 655 patients with abdominal aortic aneurysms is an unpublished ongoing study of consecutively diagnosed abdominal aorta aneurysm patients from the department of clinical genetics at the Erasmus MC in Rotterdam.

**Genotyping**

**NGS of our Parkinson’s cohort:**

DNA was isolated according to standardized procedures using the QIAsymphony DSP DNA Midi Kit (Qiagen). The DNA concentration of the samples was determined using Picogreen® (Invitrogen) measurement prior to amplification. A region of 7050 base pairs (bp) spanning the entire *GBA1* gene (11 exons and intervening introns, based on NCBI reference NM_000157.3) was amplified using long-range PCR (TaKaRa LA Taq DNA Polymerase Hot-Start Version: RR042B), with forward read primer GTTGTCACCCATACATGCCC (unique to functional gene) and reverse read primer CTCTCATGCATTCCAGAGGC (not unique to functional gene), to avoid the pseudogene being co-amplified. These primers were previously used to amplify the *GBA1* gene unambiguously (1).

Subsequently, the long-range PCR product was fragmented using the Bioruptor Pico (Diagenode) to an average size of 300 – 500 bp before sequencing on an Illumina sequencer. Library preparation was performed using the NEBNext® Ultra II DNA Library Prep kit (New England Biolabs E7370S/L). End repair/A-tailing, ligation of sequencing adapters and PCR amplification was performed according to the procedure described in the NEBNext® Ultra DNA Library Prep kit instruction manual. The quality and yield of the library preparation was determined by Fragment Analyzer analysis. Clustering and DNA sequencing (paired-end 150 bp) using the Illumina cBot and HiSeq 4000 was performed according to the manufacturer's protocols. Image analysis, base calling and quality check was performed with the Illumina data analysis pipeline RTA v2.7.7 and Bcl2fastq v2.17.

Data analysis was performed using a standardized in-house pipeline developed by GenomeScan B.V., based on the Genome Analysis Toolkit’s (GATK) best practice recommendations (2), including instructions for raw data quality control, adapter trimming, quality filtering, alignment of short reads, and frequency calculation. During the alignment step (using Burrows-Wheeler Aligner v0.7.4) to the human reference (hg19), the *GBAP1* gene was masked due to the high homology with *GBA1*. By masking *GBAP1*, mapping quality of *GBA1* reads increased, especially at the 3’-prime of the gene, where the homology is the highest.

**PacBio post-hoc analysis.**

Three samples were reassessed: one with p.D140H and p.E326K, one with p.T369M and p.L444P, and one with a possible recombination.

GBA1 amplicons were amplified as described for Illumina sequencing.

According to PacBio protocols the DNA samples were prepared for sequencing. Concentration of the samples was determined using the Qubit fluorometer and based on the concentration the samples were normalized to the required input.

Additional purification of GBA1 amplicons was performed with an AmpureXP bead ratio of 0.6x. SMRTbell (hairpin adapter ligated circular DNA molecule) library prep performed using the Template Prep kit 1.0 from PacBio. The library prep workflow contained the following steps: DNA Damage repair, End repair (blunt-end), ligation of PacBio barcoded hairpin adapters and a final exonuclease (III and VII) treatment to remove non-ligated products leaving only intact SMRTbells. Finally, all samples were purified by 3 rounds of AmpureXP bead purification and were eluted in a final volume of 15 µL EB buffer (10mM Tris-HCL pH 8). The size and concentration of the SMRTbells was determined using the Fragment Analyzer Large Fragment HS 50kb kit (Agilent) and the Qubit fluorometer (Invitrogen). Based on the DNA concentration, samples were combined in equal amounts into a single pool.

SMRT Link software (version 5.1.0.24551) was used to prepare the pool of samples for diffusion loading on a SMRTcell 1M sequencing chip. Following the instructions of SMRT Link Sample Setup the sequencing primer (version 4) and polymerse (version 2.1) were bound to the SMRTbell to form a SMRTbell complex. The complex was purified using AmpurePB beads (PacBio Ampure beads) to remove excess primers and polymerase. Finally, a PacBio Control Complex (version 2.1) was added according to SMRT Link Sample Setup instructions. In SMRT Link Run Design the sequencing run was setup with the following parameters; insert size set at 5000 bp, movie time 10 hours, immobilization time 2 hours, pre-extension time 2 hours.

*SMRT Analysis*

CCS reads were generated and mapped to the human reference Hg19 using SMRT Link. The mapped reads were visualized in IGV (version 2.4.9). To have a clean view of the PacBio reads, the insertion/deletions were hidden using Quick Consensus mode. By grouping the reads on variants, phased alleles could be visualized and the known mutations confirmed.

**Sanger sequencing validation:**

For the first 138 samples with an exonic *GBA1* variant, exon 8 was resequenced with Sanger sequencing using the standard methodology of the Amsterdam University Medical Centers for GD screening (3), to confirm the results and validate the NGS methodology for *GBA1* used in this study.

In order to avoid amplification of the pseudogene, PCR amplification for Sanger sequencing was performed in a two-step reaction. First a long fragments  encompassing exons 8-11 was amplified by PCR. This was followed by a nested PCR for exon 8. PCR fragments were treated with SAP-EXO nuclease and sequenced.  Primer sequences are available upon request.

**Genotype classification**

Genotypes were classified into four categories, based on clinical associations:

1. “GD”, for an allele reported in at least a single GD case, either in a homozygous state or in a compound heterozygous state with other GD-associated variants;
2. ii) “PD”, for an allele associated with PD or reported in PD patient(s), but not in GD;
3. iii) Synonymous, if no amino acid change was induced; or
4. iv) Novel, if a variant was not reported before.

The Human Gene Mutation Database (HGMD) was accessed to retrieve the associated phenotype and the reference was cross-checked to confirm the genotype-phenotype association.

If a subject had both a known and a novel variant, the genotype was considered novel for analysis.

For classification of subjects with multiple known variants with an unknown phase, the severest variant was leading for analysis. This was chosen because it was considered most likely that a deleterious effect of a variant would not be negated by an additional known milder variant.

**Splice site variant assessment:**

All variants, including synonymous and intronic variants, that were 6 nucleotides or closer to a splice site, were assessed with four in silico splicing programs implemented in Alamut (Alamut Visual version 2.13; Interactive Bosoftware, Rouen, France). If at least 3 out of 4 programs predicted a splicing efficiency decrease of more than 10%, this variant was considered to possibly affect splicing.

**Method cross-validation with control cohorts**

For the cross validation of the control cohort method (WES), PD samples were used with a confirmed variant. Firstly, 24 PD samples with the p.E326K variant and 24 PD samples with the p.D140H+p.E326K complex allele were repeated with WES, to assess sensitivity (assess risk of false negatives). Secondly, samples of the control cohort with a non-synonymous *GBA1* variant were repeated with our full *GBA1* NGS method, to confirm all variants found in controls (exclude false positives).

**WES sequencing of the AAA control cohort:**

Written informed consent was provided for use of genetic data for genetic research. For the AAA control cohort, WES was performed at Erasmus MC (Biomics), using an Illumina HiSeq2500 or HiSeq4000 sequencer. Of these samples, 464 were enriched using Agilents HaloPlex Target Enrichment System. The other 181 samples were enriched for clinically relevant exons using the Agilent Technologies SureSelect Clinical Research Exome (CRE) capture kit. Reads were aligned to the reference genome (hg19) using BWA, and variants were called using the GATK software. Annotation was done using Annovar3 .

The average whole exome coverage was >90 times, with 90% of the target regions covered more than 20 times. The average coverage of the *GBA1* gene (coding exons, and intronic regions including 5 bp flanking the splicing sites) was 101 times, of which 80% was covered more than 10 times. All exonic variants were considered.

**Family history**

For familial aggregation, participants were asked how many family members they have and how many of these have been diagnosed with Parkinson’s disease. In order to increase reliability of the response, this was asked separately for children, siblings, parents, grandparents, aunts/uncles and first cousins. For the analyses, these data were combined to total first- and second-degree relatives. Patients with *GBA1* wildtype and with p.E326K, p.N370S, p.L444P or p.D140H+p.E326K were included, in order to compare these genotype groups. For the possible founder location of p.D140H+p.E326K, participants were asked where their parents and grandparents were born.

For participants that indicated that a family member had symptoms of Parkinson’s disease, but they were not sure of a diagnosis of Parkinson’s disease, these family members were not designated as having Parkinson’s disease.

## Supplementary Results

**Sanger validation of NGS method (of PD cohort)**

As per our cross-validation approach, of the first 138 samples identified with a *GBA1* variant, the variants within exon 8 (1x p.L324P, 79x p.E326K, 32x p.T369M) were confirmed using Sanger sequencing.

***GBA1* variants**

The novel variant p.D453L consisted of 2 changed nucleotides within the same codon. Two novel variants were only seen combined with another known variant, however with an uncertain phasing (unknown whether these variants are on the same allele or on different alleles) of both variants (p.S-1T with p.T369M; p.G390E with p.E326K). Three different likely recombinant alleles were identified, of which two are known (1x p.D409+p.L444P+p.A456P+p.V460=, a.k.a. RecTL; 4x p.L444P+p.A456P+p.V460=, a.k.a. RecNciI) and one is novel (1x p.L268=+p.S271G+p.D409H), in six subjects.

Six homozygous or compound heterozygous variants were identified in 14 subjects (Table 1):

| **Allelic names** | Clinical association | Therefore classified as in analysis: |
| --- | --- | --- |
| L324P / T369M | GD / PD | GD |
| D140H+E326K / T369M | GD / PD | GD |
| D140H+E326K / E326K | GD / PD | GD |
| E326K / T369M | PD / PD | PD |
| E326K / E326K | PD / PD | PD |
| T369M / T369M | PD / PD | PD |

Twelve genotypes with multiple variants were identified in 14 subjects, for which we could not assess their phase due to distant genetic location. These could either have complex alleles or be compound heterozygous (Table 1).

| **Allelic names** | Clinical association | Therefore classified as in analysis: |
| --- | --- | --- |
| **S-1T, T369M** | Novel, PD | Novel |
| **Q-7R, N370S** | Novel, GD | Novel |
| **D140H+E326K, V459=** | GD, Syn | GD |
| **D140H+E326K, R496H** | GD, GD | GD |
| **R170H, E326K** | Novel, PD | Novel |
| **E326K/T369M, L444P** | PD / PD, GD | GD |
| **E326K, G390E** | PD, Novel | Novel |
| **E326K, V459=** | PD, Syn | PD |
| **E326K, V460=** | PD, Syn | PD |
| **T369M, D453L** | PD, Novel | Novel |
| **T369M, L444P** | PD, GD | GD |
| **N370S, L444P** | GD, GD | GD |

*PacBio post-hoc analysis.*

Three samples were reassessed: one with p.D140H and p.E326K, one with p.T369M and p.L444P, and one with a possible recombination. These resulted in p.D140H+p.E326K/wildtype, p.T369M+p.L444P/wildtype and Recombinant/wildtype.

**WES Control cohort validation**

In the control cohort (WES), 46 samples had a suspected non-synonymous *GBA1* variant and 2 samples had a suspected synonymous variant. Only samples with a suspected non-synonymous variant were rerun using NGS for confirmation. Two samples (p.L324P; p.T369M) did not have enough left-over material for a rerun (as a conservative approach these were considered to be true-positives). Four samples with p.L444P could not be confirmed, probably due to a false-positive call of WES due to the *GBA1* pseudogene. This means 42 samples were considered to have a non-synonymous *GBA1* variant. In three samples with p.E326K according to WES, an additional p.D140H variant was found using NGS. Upon reassessment of the control WES raw data, these three p.D140H variants were detected, but did not pass standard QC/filtering, likely due to local low coverage.

After rerunning 24 PD samples with p.E326K (one homozygous) and 24 samples with p.D140H+p.E326K using WES, all variants were confirmed. One p.D140H variant had a variant frequency of less than 30% of the reads, which would result in a non-call when using the standard heterozygous variant calling filter at 30%.

**Familial aggregation of PD and GBA variants**

See supplementary figure 2 and supplementary table 2. The effect on familial aggregation for PD was largest in the p.D140H+p.E326K group (5.6%, 21/376), which was significantly larger than for p.E326K (3.1%, 31/1014; OR 1.8, 95% CI: 1.1-3.1, p=0.037) and for *GBA1* Wildtype (2.0%, 15/736; OR 2.7, 95% CI: 1.4-5.3, p=0.003). Of all carriers, 30.1% had at least one first or second degree relative with PD, compared to 20.0% of wildtypes (OR 1.8, 95% CI: 1.0-2.9; p=0.049). Two subjects with p.E326K and one subject with p.N370S indicated a first-degree relative with Lewy body dementia, these were included for PD familial aggregation.

**Founder location p.D140H+E326K**

The p.D140H+p.E326K complex allele was seen relatively often in participants from northern Netherlands (37/995 = 3.7%, recruited through University Medical Centre Groningen and surrounding hospitals, in the northern Netherlands). Supplementary figure 3 shows a heat maps of the Netherlands, in which the first map indicates how many patients were recruited with their residence per province and the second indicates the grandparent place of birth of all p.D140H+p.E326K carriers, corrected for total number recruited per province. Based on visual inspection (no formal statistical testing), this analysis suggests the northern Netherlands as a possible founder location for this complex allele.

## Supplementary Discussion

**Study limitations**

Average age of the control cohorts was similar, but neurological status was unknown, so it is possible that some control subjects might have PD. The prevalence of PD in people over the age of 60 is approximately 1% (4). If a wildtype control had PD, impact on our results would be negligible. If a control with a *GBA1* variant had PD, the current impact would be an underestimation of the effect size of that variant.

Age at diagnosis was 3.7 years lower in subjects with a *GBA1* variant. Despite a possible earlier average onset in *GBA1* variant carriers, it should be noted that the variability in age at diagnosis is still large. In our cohort the age range for p.E326K carriers was 28-84 years, for Gaucher variant carriers this was 32-83 years and for *GBA1* wildtypes this was 27-92 years. Varying findings regarding average age at onset might partly be explained by different compositions of variant types per cohort. Carriers of p.L444P, considered one of the more severe variants associated with GD, have an average age at diagnosis of 52 years (range 31-65) in our cohort. It seems plausible there is a “dose-effect” based on variant severity, but this is challenging to confirm considering the rarity of a specific variant per study, together with a large variability.

Subjects self-reported that diagnosis of PD was set by a neurologist, however 81% of cases were also referred by a neurologist. Participants were explicitly asked for the name of their neurologist and when the diagnosis PD was made, to increase certainty of adequate inclusion. Because the age at diagnosis was registered, age of onset was likely several years earlier. Also, it cannot be excluded that some participants came from the same family. Although at least 15% of cases were referred by a tertiary medical center, which might skew the population to more rare PD genotypes, the majority of the cohort still represents a general population. However, the proportion of *GBA1* variants in subjects referred from a university medical center versus a non-university medical center, was indeed higher in subjects from a tertiary medical center (see Supplementary figure 4). Finally, the current design did not allow for further clinical characterization of patients.

## Supplementary table

| Demographics | | | | | | |
| --- | --- | --- | --- | --- | --- | --- |
|  | *GBA1* carriers | n **^a^** | *GBA1* non-carriers | n **^a^** | p-value | Control (n=655) |
| Age at inclusion (years ± SD (range)) | 65.1 ± 9.0 (35-87) | 510 | 68.4 ± 9.1 (34-97) | 2885 | **<0.001** | 67.2 ± 10.1 |
| Sex  (% men : women) | 62 : 38 | 507 | 65 : 35 | 2876 | 0.268 | 77 : 23 |
| Age at diagnosis (years ± SD (range)) | 56.9 ± 10.4 (25-84) | 312 | 60.6 ± 10.7 (27-92) | 1849 | **<0.001** | - |
| Early vs Late onset  (% ≤50:>50 years) | 27.2 : 72.8 | 312 | 18.2 : 81.8 | 1849 | **<0.001** | - |
| Duration of disease ^b^ | 7.1±6.0 (0-30) | 312 | 7.0 ± 6.1 (0-48) | 1849 | 0.693 | - |

Table 1 Demographics and certain Parkinson’s disease characteristics, comparing Parkinson’s disease patients with a non-synonymous *GBA1* variant to those without. Differences between these groups were tested for statistical significance. A p-value is given. Age and sex distribution are also given for the control cohort.
^a^ Number of available subjects.
^b^ Duration of disease based on date of diagnosis to date of inclusion.

| Familial aggregation in GBA1 variant carriers and non-carriers | | | | |
| --- | --- | --- | --- | --- |
|  | *GBA1* carriers | *GBA1* non-carriers | OR (95% CI) | p-value |
| First-degree relatives with Parkinson’s disease, % (n) | 3.6 (61/1689) | 2.0 (15/736) | 1.8 (1.0-3.2) | **0.043** |
| At least one first or second-degree relative with Parkinson’s disease, % (n) | 30.1 (91/302) | 20.0 (23/115) | 1.7 (1.0-2.9) | **0.049** |

Table 2 Familial aggregation compared between Parkinson’s disease carriers of a non-synonymous GBA1 variant and those without. First-degree relatives are excluding children.

| **Variant comparison with a Dutch and European (non-Finnish) Whole Genome Sequenced cohort** | | | | | | | | | | |
| --- | --- | --- | --- | --- | --- | --- | --- | --- | --- | --- |
| **Variant info** | | **Allele frequency** | | | **Heterozygous** | | | **Homozygous** | | |
| **Position**  **Chr 1** | **Allelic Name** | **Current cohort** | **GoNL** | **GnomAD (v3)** | **Current cohort** | **GoNL** | **GnomAD (v3)** | **Current cohort** | **GoNL** | **GnomAD (v3)** |
| (GRCh37/  hg19) |  | **Dutch** | **Dutch** | **EU (non-finnish)** | **Dutch** | **Dutch** | **EU (non-finnish)** | **Dutch** | **Dutch** | **EU (non-finnish)** |
|  |  | **n=3402** | **n=499** | **n=~32000** | **n=3402** | **n=499** | **n=~32000** | **n=3402** | **n=499** | **n=~32000** |
| 155210876 | **E-30Gfs*8** | 0.015% | NR | 0.005% | 0.029% | NR | 0.009% | 0% | NR | 0% |
| 155210492 | **L-24S** | 0.029% | NR | 0% | 0.059% | NR | 0% | 0% | NR | 0% |
| 155210490 | **S-23G** | 0.015% | NR | 0% | 0.029% | NR | 0% | 0% | NR | 0% |
| 155210441 | **Q-7R** | 0.029% | NR | 0.003% | 0.059% | NR | 0.006% | 0% | NR | 0% |
| 155210424 | **S-1T** | 0.015% | NR | NR | 0.029% | NR | NR | 0% | NR | NR |
| 155209816 | **V17=** | 0% | NR | 0.020% | 0% | NR | 0.040% | 0% | NR | 0% |
| 155209813 | **C18*** | 0.015% | NR | NR | 0.029% | NR | NR | 0% | NR | NR |
| 155209752 | **R39C** | 0.015% | NR | 0.009% | 0.029% | NR | 0.019% | 0% | NR | 0% |
| 155209732 | **S45Rfs*15** | 0.015% | NR | NR | 0.029% | NR | NR | 0% | NR | NR |
| 155209684 | **T61=** | 0.015% | NR | NR | 0.029% | NR | NR | 0% | NR | NR |
| 155208422 | **I119=** | 0.073% | 0.100% | 0.073% | 0.147% | NR | 0.139% | 0% | NR | 0.003% |
| 155208421 | **R120W** | 0.073% | NR | 0.003% | 0.147% | NR | 0.006% | 0% | NR | 0% |
| 155208389 | **I130=** | 0.015% | NR | NR | 0.029% | NR | NR | 0% | NR | NR |
| 155208361 | **D140H** | 1.264% | NR | 0.036% | 2.528% | NR | 0.071% | 0% | NR | 0% |
| 155208350 | **Q143=** | 0.015% | NR | 0.008% | 0.029% | NR | 0.015% | 0% | NR | 0% |
| 155208060 | **R170H** | 0.029% | NR | 0.003% | 0.059% | NR | 0.006% | 0% | NR | 0% |
| 155208001 | **A190T** | 0.015% | NR | NR | 0.029% | NR | NR | 0% | NR | NR |
| 155207990 | **G193=** | 0.015% | NR | 0.003% | 0.029% | NR | 0.006% | 0% | NR | 0% |
| 155207984 | **G195=** | 0.015% | NR | 0.002% | 0.029% | NR | 0.003% | 0% | NR | 0% |
| 155207965 | **G202R** | 0.015% | NR | 0.003% | 0.029% | NR | 0.006% | 0% | NR | 0% |
| 155207374 | **c.762-5G>A** | 0.015% | NR | NR | 0.029% | NR | NR | 0% | NR | NR |
| 155207367 | **F216Y** | 0.015% | 0.100% | 0.003% | 0.029% | NR | 0.006% | 0% | NR | 0% |
| 155207266 | **G250S** | 0.015% | NR | NR | 0.029% | NR | NR | 0% | NR | NR |
| 155207249 | **H255Q** | 0.029% | NR | 0.028% | 0.059% | NR | 0.056% | 0% | NR | 0% |
| 155207235 | **I260T** | 0.029% | NR | NR | 0.059% | NR | NR | 0% | NR | NR |
| 155207210 | **L268=** | 0.015% | NR | NR | 0.029% | NR | NR | 0% | NR | NR |
| 155207203 | **S271G** | 0.015% | NR | NR | 0.029% | NR | NR | 0% | NR | NR |
| 155206172 | **L324P** | 0.029% | NR | 0.003% | 0.059% | NR | 0.006% | 0% | NR | 0% |
| 155206170 | **G325R** | 0.015% | NR | NR | 0.029% | NR | NR | 0% | NR | NR |
| 155206167 | **E326K** | 4.718% | 2.300% | 1.283% | 9.024% | NR | 2.530% | 0.206% | NR | 0.019% |
| 155206158 | **R329C** | 0.029% | NR | NR | 0.059% | NR | NR | 0% | NR | NR |
| 155206111 | **G344=** | 0.015% | NR | 0.002% | 0.029% | NR | 0.003% | 0% | NR | 0% |
| 155206101 | **W348G** | 0.015% | NR | 0% | 0.029% | NR | 0% | 0% | NR | 0% |
| 155206093 | **Q350H** | 0.015% | NR | 0.008% | 0.029% | NR | 0.015% | 0% | NR | 0% |
| 155206037 | **T369M** | 1.470% | 1.100% | 0.951% | 2.881% | NR | 1.896% | 0.029% | NR | 0.003% |
| 155206036 | **T369=** | 0.029% | NR | 0.022% | 0.059% | NR | 0.043% | 0% | NR | 0% |
| 155205634 | **N370S** | 0.470% | NR | 0.181% | 0.941% | NR | 0.363% | 0% | NR | 0% |
| 155205619 | **V375G** | 0.015% | NR | NR | 0.029% | NR | NR | 0% | NR | NR |
| 155205605 | **D380Y** | 0.015% | NR | NR | 0.029% | NR | NR | 0% | NR | NR |
| 155205581 | **E388K** | 0.044% | NR | 0.025% | 0.088% | NR | 0.050% | 0% | NR | 0% |
| 155205574 | **G390E** | 0.015% | NR | NR | 0.029% | NR | NR | 0% | NR | NR |
| 155205568 | **N392S** | 0.015% | NR | 0% | 0.029% | NR | 0% | 0% | NR | 0% |
| 155205518 | **D409H** | 0.044% | NR | 0.017% | 0.088% | NR | 0.034% | 0% | NR | 0% |
| 155205043 | **L444P** | 0.456% | 0.401% | NR | 0.911% | NR | NR | 0% | NR | NR |
| 155205018 | **P452=** | 0.015% | NR | 0.003% | 0.029% | NR | 0.006% | 0% | NR | 0% |
| 155205017 | **D453H** | 0.059% | NR | 0.003% | 0.118% | NR | 0.006% | 0% | NR | 0% |
| 155205016 | **D453V** | 0.059% | NR | 0.003% | 0.118% | NR | 0.006% | 0% | NR | 0% |
| 155205008 | **A456P** | 0.073% | NR | 0.031% | 0.147% | NR | 0.062% | 0% | NR | 0% |
| 155204997 | **V459=** | 0.073% | NR | 0.029% | 0.147% | NR | 0.059% | 0% | NR | 0% |
| 155204996 | **V460M** | 0.015% | NR | 0% | 0.029% | NR | 0% | 0% | NR | 0% |
| 155204994 | **V460=** | 0.103% | NR | 0.067% | 0.206% | NR | 0.134% | 0% | NR | 0% |
| 155204986 | **R463P** | 0.029% | NR | NR | 0.059% | NR | NR | 0% | NR | NR |
| 155204829 | **S484L** | 0.015% | NR | NR | 0.029% | NR | NR | 0% | NR | NR |
| 155204818 | **S488T** | 0.015% | NR | NR | 0.029% | NR | NR | 0% | NR | NR |
| 155204811 | **H490R** | 0.015% | NR | NR | 0.029% | NR | NR | 0% | NR | NR |
| 155204793 | **R496H** | 0.015% | NR | NR | 0.029% | NR | NR | 0% | NR | NR |

Table 3 Comparison of all exonic and splice site variants in the current PD cohort to the open access GoNL and GnomAD (v3) databases. Both GoNL (coverage: ~12) and GnomAD (v3) (coverage: ~30) are whole genome sequenced databases, not specifically designed for sequencing the GBA1 gene. Because phasing of variants is not determined in these databases, all variants are given individually. This means e.g. the number of p.E326K carriers is a lot higher, because it combines all complex alleles as well, like the in our Dutch PD cohort common p.D140H+E.326K variant. All values are given in percentages. The Blue columns indicate the allele frequencies, the Yellow columns indicate the heterozygous carriers, the Orange columns indicate the homozygous carriers. GnomAD (v3) does not report the well-described p.L444P variant, because it failed GATK Allele-Specific Variant Quality Recalibration. EU=European. NR=not reported. GnomAD also contains values of 0%, because the variant was described in other populations than European (non-Finnish).

| #SSF: SpliceSiteFinder-like; MaxEnt: MaxEntScan | | |  |  |
| --- | --- | --- | --- | --- |
| ## The splicing prediction outcome displayed in this table is generated with Alamut Visual version 2.13 (Interactive Bosoftware, Rouen, France) | | | | |
| ### Green: increased affinity; Red: decreased affinity (10+% change only) | | |  |  |
| *Variant identified within CHDR and AAA cohorts | | |  |  |
| ^Variant identified within AAA cohort ONLY | |  |  |  |
|  |  | ***GBA* (NM_000157; NP_000148)** | | |
|  | **Slicing Prediction Method [Range], Treshold** | **Wild Type** | **Mutated** | **Genomic Coordinate [GRCh37/ hg19]** |
| **Variant No.1** |  | **c.112T p.Ser38 [p.S-1]** | **c.112T>A p.(Ser38Thr) [p.S-1T]** | **chr1:155210424:A:T** |
|  | SSF [0-100], ≥70 | = 95.64 | = 95.64 |  |
|  | MaxEnt [0-12], ≥0 | = 10.67 | = 10.67 |  |
|  | NNSPLICE [0-1], ≥0,4 | = 1.00 | = 1.00 (+0.1%) |  |
|  | GeneSplicer [0-15], ≥0 | = 9.29 | = 9.82 (+5.7%) |  |
|  |  |  |  |  |
| **Variant No.2** |  | **c.762-5G** | **c.762-5G>A** | **chr1:155207374:C:T** |
|  | SSF [0-100], ≥70 | = 93.20 | = 93.20 |  |
|  | MaxEnt [0-12], ≥0 | 10,84 | 10.95 (+1.0%) |  |
|  | NNSPLICE [0-1], ≥0,4 | 0,98 | 0.98 (+0.1%) |  |
|  | GeneSplicer [0-15], ≥0 | 9,63 | 10.21 (+6.1%) |  |
|  |  |  |  |  |
| **Variant No.3** |  | **c.764T p.Phe255 [p.F216]** | **c.764T>A p.(Phe255Tyr) [p.F216Y]** | **chr1:155207367:A:T** |
|  | SSF [0-100], ≥70 | = 93.20 | = 93.20 |  |
|  | MaxEnt [0-12], ≥0 | 10,84 | 11.25 (+3.8%) |  |
|  | NNSPLICE [0-1], ≥0,4 | 0,98 | 0.98 (+0.5%) |  |
|  | GeneSplicer [0-15], ≥0 | 9,63 | 10.13 (+5.2%) |  |
|  |  |  |  |  |
| **Variant No.4*** |  | **c.1223C p.Thr408 [p.T369]*** | **c.1223C>T p.(Thr408Met) [p.T369M]*** | **chr1:155206037:G:A*** |
|  | SSF [0-100], ≥70 | 85 | 85.40 (+0.5%) |  |
|  | MaxEnt [0-12], ≥0 | 9,89 | 9.26 (-6.4%) |  |
|  | NNSPLICE [0-1], ≥0,4 | 1 | 1.00 (-0.0%) |  |
|  | GeneSplicer [0-15], ≥0 | 9,63 | 8.01 (-16.8%) |  |
|  |  |  |  |  |
| **Variant No.5** |  | **c.1224G p.(Thr408) [p.T369]** | **c.1224G>C p.(Thr408=) [p.T369=]** | **chr1:155206036:C:T** |
|  | SSF [0-100], ≥70 | = 85.00 | = 72.86 (-14.3%) |  |
|  | MaxEnt [0-12], ≥0 | =9.89 | = 5.94 (-39.9%) |  |
|  | NNSPLICE [0-1], ≥0,4 | = 1.00 | = 0.94 (-6.2%) |  |
|  | GeneSplicer [0-15], ≥0 | = 9.63 | = 4.73 (-50.9%) |  |
|  |  |  |  |  |
| **Variant No.6*** |  | **c.1226A p.Asn409 [N370]*** | **c.1226A>G p.(Asn409Ser) [N370S]*** | **chr1:155205634:T:C*** |
|  | SSF [0-100], ≥70 | = 76.29 | = 76.29 |  |
|  | MaxEnt [0-12], ≥0 | 8,89 | 8.83 (-0.7%) |  |
|  | NNSPLICE [0-1], ≥0,4 | 0,92 | 0.90 (-1.7%) |  |
|  | GeneSplicer [0-15], ≥0 | 11,87 | 11.31 (-4.8%) |  |
|  |  |  |  |  |
| **Variant No.7*** |  | **c.1505G p.Arg502 [p.R463P]*** | **c.1505G>C p.(Arg502Pro) [p.R463P]*** | **chr1:155204986:C:G*** |
|  | SSF [0-100], ≥70 | 81,7 | 0.00 (-81.7%) |  |
|  | MaxEnt [0-12], ≥0 | 9,35 | 3.94 (-57.8%) |  |
|  | NNSPLICE [0-1], ≥0,4 | 0,99 | 0.69 (-30.2%) |  |
|  | GeneSplicer [0-15], ≥0 | 8,24 | 4.69 (-43.1%) |  |
|  |  |  |  |  |
| **Variant No.8^** |  | **c.1000-4G** | **c.1000-4G>T** | **chr1:155206264:C:A** |
|  | SSF [0-100], ≥70 | = 94.23 | = 94.23 |  |
|  | MaxEnt [0-12], ≥0 | 10,7 | 11.01 (+2.9%) |  |
|  | NNSPLICE [0-1], ≥0,4 | 0,99 | 0.99 (+0.1%) |  |
|  | GeneSplicer [0-15], ≥0 | 9,36 | 8.91 (-4.8%) |  |

Table 4 Splice site prediction results of all variants with a distance to splice site of 6 nucleotides or less. The p.E-30Gfs*8 variant is not included because it is frameshift mutation with early termination, making a possible effect on splicing obsolete.

## References

1. Mata IF, Leverenz JB, Weintraub D, Trojanowski JQ, Chen-Plotkin A, Van Deerlin VM, et al. GBA Variants are associated with a distinct pattern of cognitive deficits in Parkinson's disease. Mov Disord. 2016;31(1):95-102.

2. McKenna A, Hanna M, Banks E, Sivachenko A, Cibulskis K, Kernytsky A, et al. The Genome Analysis Toolkit: a MapReduce framework for analyzing next-generation DNA sequencing data. Genome Res. 2010;20(9):1297-303.

3. Lesage S, Anheim M, Condroyer C, Pollak P, Durif F, Dupuits C, et al. Large-scale screening of the Gaucher's disease-related glucocerebrosidase gene in Europeans with Parkinson's disease. Hum Mol Genet. 2011;20(1):202-10.

4. Pringsheim T, Jette N, Frolkis A, Steeves TD**.** The prevalence of Parkinson's disease: a systematic review and meta-analysis. Mov Disord. 2014;29(13):1583-90.

## Supplementary figures

*Attached as TIF files.*
