# Supplementary figures and images for: A Large‐Scale Full GBA1 Gene Screening in Parkinson's Disease in the Netherlands
Source: Mov Disord. 2020 Jul 2;35(9):1667–74. doi: 10.1002/mds.28112 (PMC7540512; doi:10.1002/mds.28112)

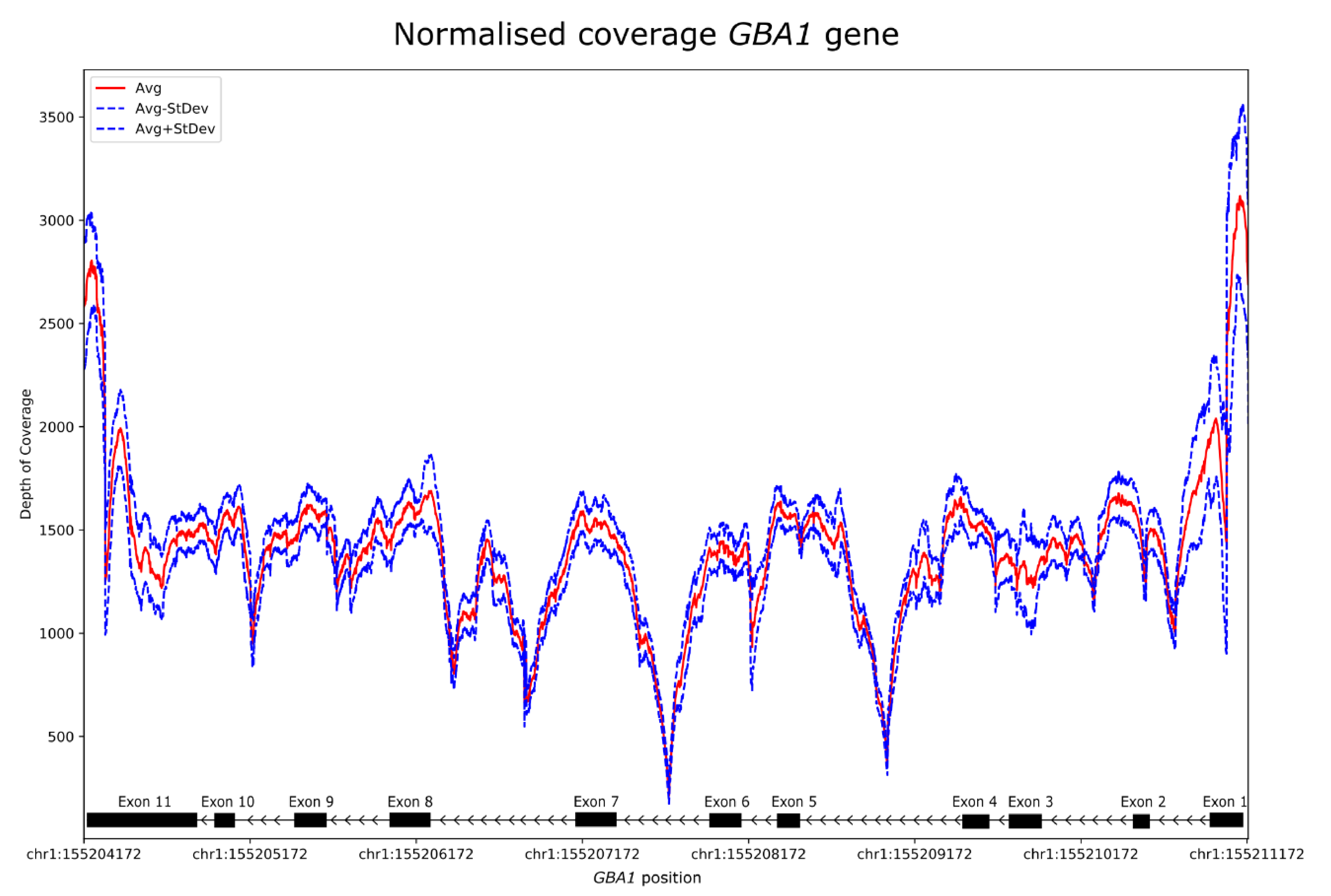

Supplement: Supplementary file 1 — Appendix S1: Supplementary data [file MDS-35-1667-s001.zip › MDS_28112_Den Heijer et al_GBA1 genotyping manuscript_Supp fig 1_30Dec19.tiff]

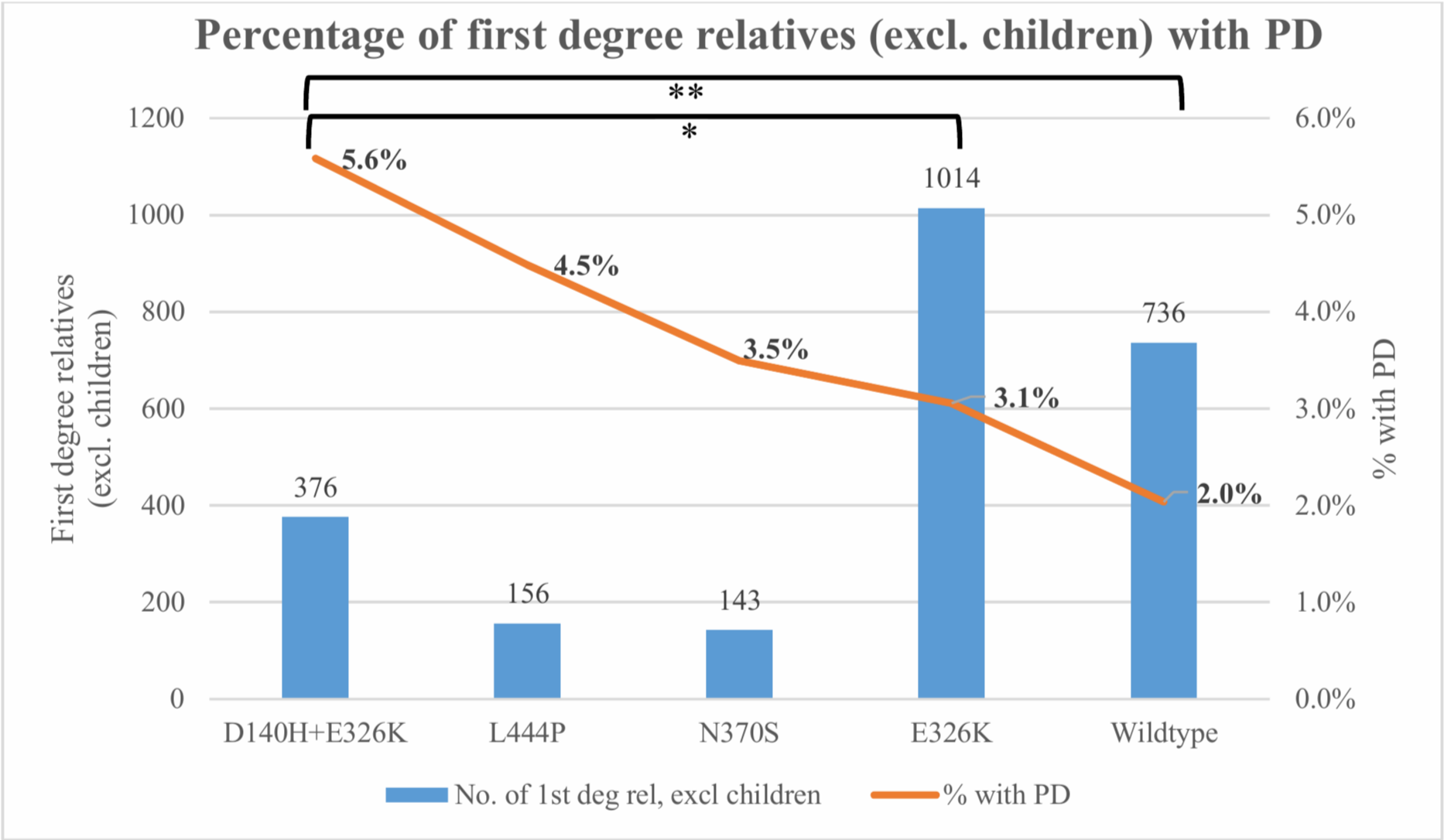

Supplement: Supplementary file 1 — Appendix S1: Supplementary data [file MDS-35-1667-s001.zip › MDS_28112_Den Heijer et al_GBA1 Genotyping manuscript_Supp fig 2_14Mar20.tiff]

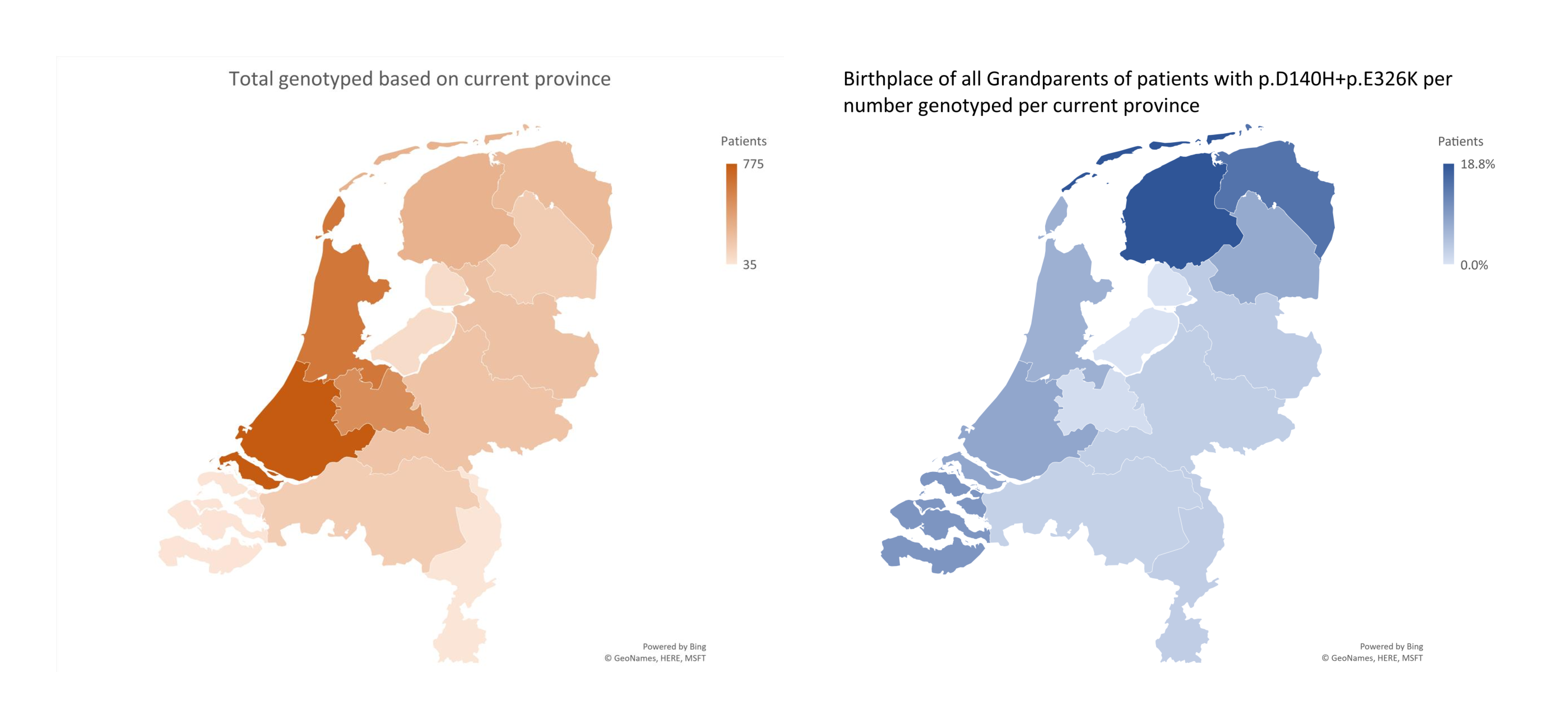

Supplement: Supplementary file 1 — Appendix S1: Supplementary data [file MDS-35-1667-s001.zip › MDS_28112_Den Heijer et al_GBA1 Genotyping manuscript_Supp fig 3_30Dec19.tiff]

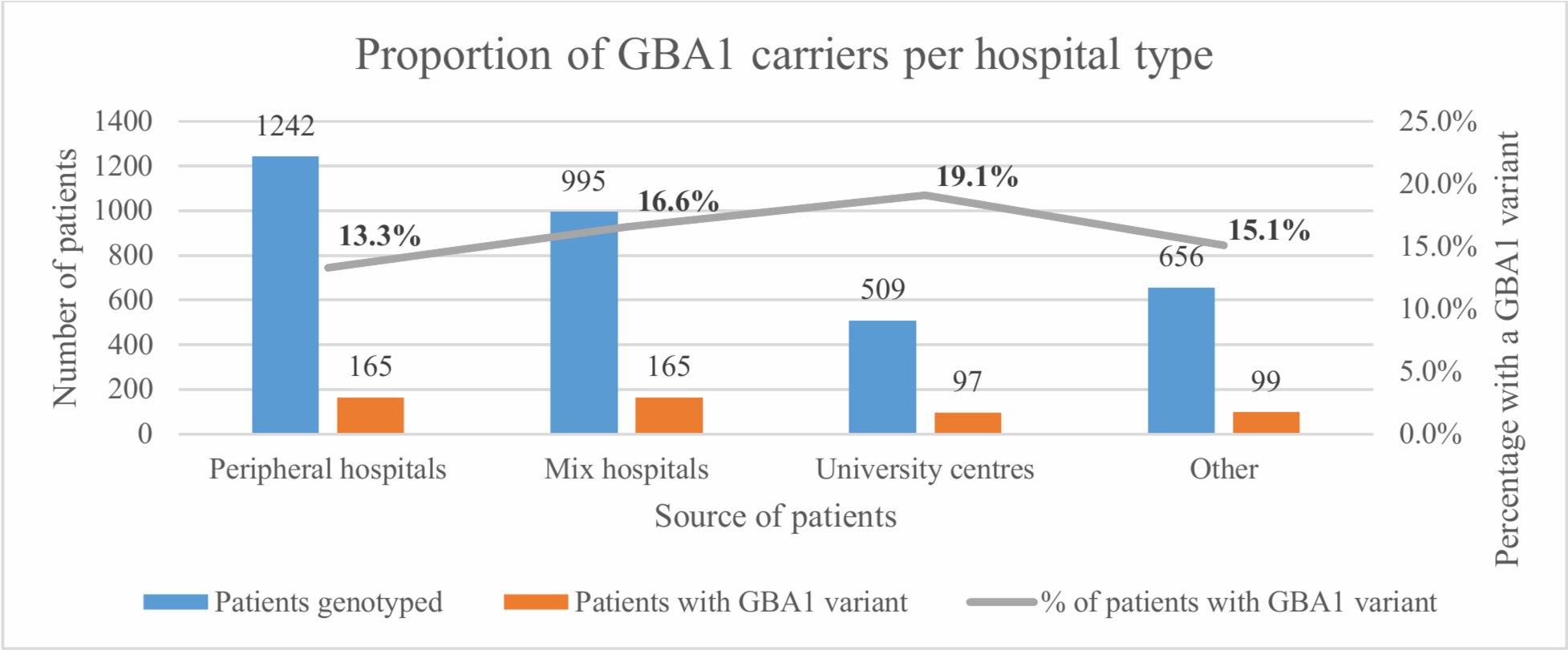

Supplement: Supplementary file 1 — Appendix S1: Supplementary data [file MDS-35-1667-s001.zip › MDS_28112_Den Heijer et al_GBA1 Genotyping manuscript_Supp fig 4_14Mar20.tiff]
